# Perspectives on Principles of Cellular Behavior from the Biophysics of Protists

**DOI:** 10.1093/icb/icad106

**Published:** 2023-07-26

**Authors:** Ben T Larson

**Affiliations:** Department of Biochemistry and Biophysics, University of California, San Francisco, CA 94158, USA

## Abstract

Cells are the fundamental unit of biological organization. Although it may be easy to think of them as little more than the simple building blocks of complex organisms such as animals, single cells are capable of behaviors of remarkable apparent sophistication. This is abundantly clear when considering the diversity of form and function among the microbial eukaryotes, the protists. How might we navigate this diversity in the search for general principles of cellular behavior? Here, we review cases in which the intensive study of protists from the perspective of cellular biophysics has driven insight into broad biological questions of morphogenesis, navigation and motility, and decision making. We argue that applying such approaches to questions of evolutionary cell biology presents rich, emerging opportunities. Integrating and expanding biophysical studies across protist diversity, exploiting the unique characteristics of each organism, will enrich our understanding of general underlying principles.

## Introduction

**Fig. 1:**
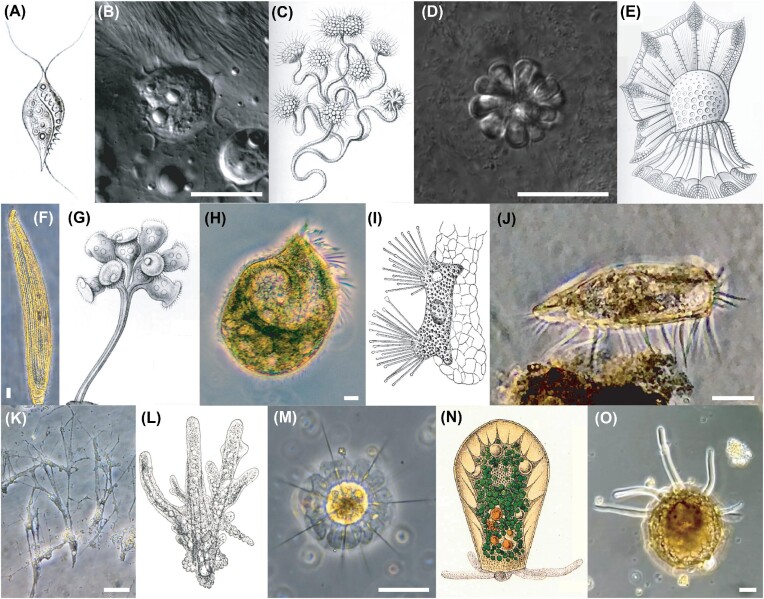
Protists display a great diversity of cellular form and function. Various protists from historical literature (drawings) and from environmental samples illustrate this diversity. (A–E) Various flagellates. (A) *Trichomonas intestinalis*, an intestinal parasite of humans (Haeckel 1904). (B) *Trichomitopsis* sp. collected from the hindgut of a termite, a gut endosymbiont that participates in cellulose digestion. (C) *Anthophysa vegetans*, often found in rivers and noted for its tendency to accumulate iron and manganese in the stalks of its colonies (Haeckel 1904). (D) Rosette colony of the choanoflagellate *Barroeca monosierra* originally isolated from a saline soda lake, which harbors a microbiome (Hake et al. 2021). (E) *Ornithocercus magnificus*, a marine dinoflagellate noted for its particularly complex morphology. Adapted from (Haeckel 1904). (F–J) Various ciliates. (F) Unidentified ciliate collected from a hypersaline splash pool with prominent cortical rows of cilia, a common feature of ciliates. (G) *Zoothamnium arbuscula*, a colonial ciliate known for its ability to rapidly contract its stalk in response to aversive stimuli and grow to macroscopically visible sizes. Adapted from (Haeckel 1904). (H) *Fabrea salina*, often a dominant protist in hypersaline environments with the ability to inhibit the growth of other halotolerant species, collected from a marine splash pool (Guermazi et al. 2008). (I) The suctorian *Trichophrya salparum* on the gill of a salp, adapted from (Calkins 1926). Suctorians begin life as swimming cells before settling on a substrate and undergoing metamorphosis into the depicted tentacled form. (J) *Euplotes* sp., known for their ability to walk using leg-like bundles of cilia, isolated from a stagnant puddle filled with dead leaves. (K–O) Various amoebae. (K) Unidentified rhizarian amoeba with a complex reticulopodial network collected from a tide pool. (L) *Amoeba proteus*, a large amoeba studied by Jennings for its complex hunting behavior. Image adapted from (Jennings 1906) after Leidy. (M) Unidentified radiolarian with its characteristic spiny, mineralized silica skeleton, collected from a near shore marine sample. (N) *Hyalosphenia papilo*, amoeba with an organic test (shell) that harbors endosymbiotic algae. Drawing adapted from (Leidy 1879). (O) *Difflugia* sp., isolated from a vernal farm pond, which has built its test from mineral particles collected by the cell from its environment. Scale bars are 25 μm.

Life is fundamentally cellular. Proper biological function emerges from the regulated interplay between subcellular processes, cellular behaviors, and physical constraints. The search for principles dictating this interplay therefore represents a major transdisciplinary challenge that requires integrative approaches. All cellular behavior takes place in the context of physical constraints such as those imposed by geometry, mechanics, and diffusive transport. By imposing limits, constraints can serve not only as barriers, but can also yield robustness of function (Ashby 1956). In this way, constraints can be mechanistically important, and physical processes can constitute mechanisms in their own rights. Combining perspectives from physics and biology has been instrumental to addressing many mechanistic questions in biology. In the context of cell biology, these include, for example, the regulation of cytokinesis (Burton and Taylor 1997; Pollard 2010; Dorn et al. 2016; Mogilner and Manhart 2016) and cell crawling (Lee et al. 1994; Mogilner and Oster 1996, 2003; Pollard and Cooper 2009). However, much of this work has been confined to a relatively small number of well-studied systems representing a small subset of eukaryotic diversity. The variety of plants and animals apparent from everyday experience belies that fact that protists, a paraphyletic group defined by the absence of the complex multicellularity found in plants, animals, and fungi, constitute the vast majority of eukaryotic diversity. The diversity, though, is reflected in sundry protistan forms, functions, and behaviors (Fig. 1).

Protists play important roles in nearly every ecosystem and also hold key phylogenetic positions for elucidating major evolutionary events, including the origin and evolution of various organelles (Gray 1989; Elde et al. 2005; Gould et al. 2008; McFadden 2014), the origin of eukaryotes (Baldauf et al. 1996; Cavalier-Smith 1997; Fritz-Laylin et al. 2010; Koonin 2010; Williams 2014), and the origin of animals (King et al. 2003; Ruiz-Trillo et al. 2008; Sebe-Pedros et al. 2011; Brunet and King 2017; Richter et al. 2018). Despite their ecological and evolutionary importance, the biology of most protists remains poorly understood (Keeling and Campo 2017; Collier and Rest 2019). In the investigation of general principles underlying the regulation and evolution of form and function in eukaryotes, protists represent a vast, largely untapped resource.

Most protists are free-living, so the natural behaviors of cells are amenable to direct observation and targeted manipulation in controlled environments. Indeed, in contrast to a modern focus on relatively few model organisms and cell lines, a substantial amount of the history of cell biology from the development of microscopy to the rise of molecular biology has been dominated by careful observation of protist behavior and structure, including some of the most extensive, detailed early observations of cells (protists described as wondrous “wee animalcules” by Antonie van Leeuwenhoek; Leeuwenhoek 1677; Leeuwenhoek and Dobell 1932; Dröscher 2014; Lane 2015; Richardson et al. 2015). Recently, coinciding with the increasing interest in nontraditional model systems, descriptive and mechanistic characterizations of protists are becoming more prevalent (Collier and Rest 2019; Faktorová et al. 2020). Although nontraditional, the development and study of these systems benefits from sometimes extensive historical documentation of cell structure, physiology, behavior, and life history. Both historical and recent work on protists illustrates how investigating function at the intersection of biology and physics can provide mechanistic insight into the emergence of organismal function. Furthermore, as the following sections of this manuscript illustrate, this perspective reveals general principles transcending the fascinating yet idiosyncratic biology of any particular protist.

**Fig. 2:**
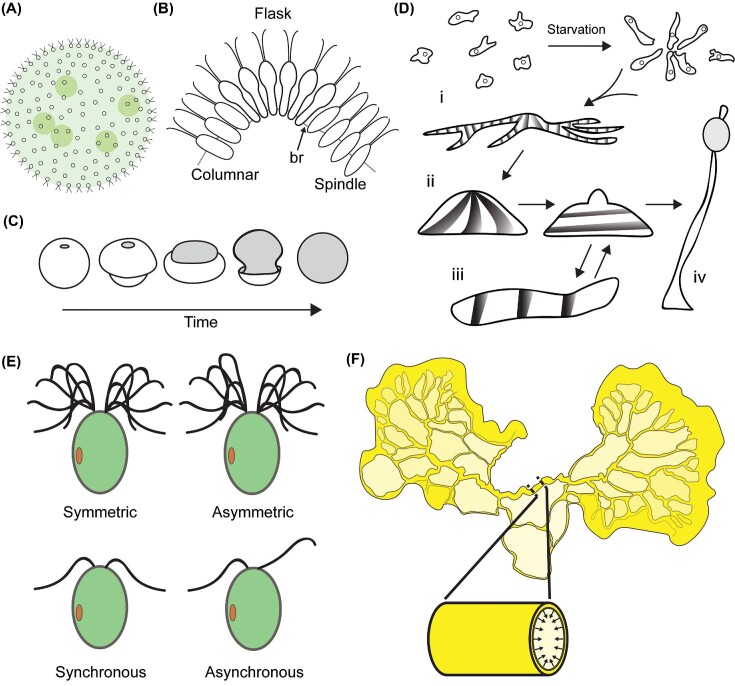
Biophysical approaches to understanding form and behavior in protist systems have led to mechanistic insight into organismal function. (A–C) Embryo inversion in the green alga *Volvox* serves as a model for morphogenesis based on the bending of cellular sheets. (A) *Volvox* forms large, spherical colonies composed of biflagellate cells connected by cytoplasmic bridges and embedded in an extracellular matrix (ECM). Colonies have differentiated germ and somatic cells, and embryos form and develop inside the colonies. (B) Developing embryos turn themselves inside out during development in a process called inversion. Inversion is driven by coordinated cell shape changes from spindle to flask to columnar cells. Cytoplasmic bridges (br) are also displaced and bridge tension ultimately leads to the bending of the sheet of cells. (C) Overview of shape change during embryo inversion. Different species of *Volvox* undergo different types of inversion processes. The most extensively studied one depicted here is known as “B” type. Gray shading represents the surface of the embryo with flagella pointing outward. Illustrations based in part on figures from (Haas and Goldstein 2018). (D) *Dictyostelium* morphogenesis under starvation conditions is controlled by reactiondiffusion pattern formation stemming from cAMP activity. Arrows indicate the progression of the starvation response from aggregation of amoeboid cells (i) leading to mound formation (ii) to slug (iii) and/or stalk formation (iv). In each stage, waves of cAMP activity, indicated in gray, orchestrate cellular behaviors including directed migration, ECM secretion, adhesion, and differentiation in order to robustly and reproducibly form the multicellular structure. Based in part on figures and information from (Dormann et al. 2002; Weijer 2004; Sriskanthadevan et al. 2011). (E) The green alga *Chlamydomonas* serves as a model for cellular motility and navigation in the context of fluid mechanics. The top images depict flagellar wave forms of the biflagellate *Chlamydomonas* cell. Bottom images indicate synchronous and asynchronous flagellar dynamics, which play a role in phototaxis. Light is sensed by an eyespot (red spot). Differential flagellar response in terms of flagellar beat amplitude (asymmetric) due to light sensing allows the cell to turn towards light when undergoing phototaxis (Bennett and Golestanian 2015; Leptos et al. 2023). Flagellar waveforms based in part on (Geyer et al. 2013). (F) The slime mold *Physarum polycephalum* is a large, multinucleate cell, up to centimeters in size, organized as a network of contractile tubes with dynamic structure through which cytoplasm is pumped. Due to its ability to select optimal paths connecting resources in complex environments, *Physarum* has long served as a model for cellular decision-making.

Here, we review representative cases in which the intensive study of protists from the perspective of cellular biophysics has driven insight into the general, interrelated biological problems of morphogenesis, navigation and motility, and decision making. Sections concerning these problems focus primarily on some of the best-developed respective protist systems: the colonial green alga *Volvox*, which undergoes multicellular morphogenesis from an embryo stage and the social amoeba *Dictyostelium*, which can undergo an elaborate multicellular morphogenetic progression as part of its life cycle; the unicellular alga *Chlamydomonas* and multicellular *Volvox*, which both exhibit phototactic behavior; and the multinucleate slime mold *Physarum*, noted for its complex foraging behavior (Fig. 2). We argue that applying such biophysical perspectives to questions of evolutionary cell biology presents a wealth of emerging opportunities. Expanding biophysical studies and integrating them with complementary approaches across protist diversity, exploiting characteristics of each organism that uniquely highlight general biological questions, will enrich our understanding of underlying principles.

## Protist behavior from a biophysical perspective

### Morphogenesis

Cellular behavior in the context of morphogenesis has perhaps been most extensively studied from the perspective of biophysics. D'Arcy Thompson, in his pioneering “On Growth and Form,” pointed out that problems of growth and morphology are essentially mathematical and physical problems (Thompson 1917). This work is often identified as the first to bring ideas from mathematics and physics to bear on problems of biological morphogenesis. Although most are unicellular, various protists form multicellular colonies according to diverse morphogenetic processes. The relative simplicity of multicellular morphogenesis in protists can serve to clarify the roles of cellular behaviors and physical mechanisms.

The green alga *Volvox*, with its large, swimming colonies produced by a developmental process has long captivated microscopists (Leeuwenhoek 1700; Fig. 2A). Like embryogenesis in many animals, *Volvox* colony development begins with an early embryogenesis phase of synchronous cleavage divisions, during which cells divide without growth (Matt and Umen 2016). This early phase is followed by a growth and expansion phase mediated by ECM secretion (Ueki and Nishii 2009; Matt and Umen 2016). After the growth phase, nascent colonies undergo an inversion process, in which the entire colony flips inside out (Viamontes and Kirk 1977; Matt and Umen 2016) (Fig. 2B and C). This inversion process is reminiscent of gastrulation in many animals in that such processes involve the bending of sheets of cells through coordinated cellular deformations. In *Volvox*, inversion allows cells to reorient their flagella from pointing toward the inside of the spherical colony to the outside in order for the colony to swim.

While qualitative descriptions of the dynamics of inversion have existed for decades (Viamontes and Kirk 1977), recent advances in microscopy have facilitated detailed, quantitative analysis (Höhn et al. 2015; Haas et al. 2018). Dynamics alone, however, only provide the starting point for an explanation. As in any multicellular morphogenetic process, correlations between local cellular behavior and global, organism scale, deformations can be observed (Fig. 2A and B), but determining precisely how those cellular behaviors are related to the global shape changes requires further investigation. For example, whether cellular deformations are the cause or the result of tissue bending cannot necessarily be determined purely by observation. Understanding the mechanical aspects of shape changes can help answer such questions. Mathematical modeling together with quantitative analysis of *Volvox* inversion has shown how coordinated cell shape changes (Fig. 2B) along with geometrical and mechanical constraints inherent to elastic bending drive the inversion process (Höhn et al. 2015; Haas et al. 2018). Cells in *Volvox* colonies are connected by cytoplasmic bridges that constitute a stable, structural framework that physically couples all cells (Viamontes and Kirk 1977; Matt and Umen 2016; Fig. 2B). The details of the different phases of inversion, which differ between species, have been described in depth (Viamontes and Kirk 1977; Cole and Reedy 2003; Matt and Umen 2016; Haas et al. 2018). Briefly, inversion involves the formation of cells that are constricted on the side facing into the colony, called flask cells (Fig. 2B). Flask cells are analogous in form and function to bottle cells of animal epithelia, which also exhibit polarized constriction and are early drivers of tissue bending during gastrulation (Viamontes and Kirk 1977; Matt and Umen 2016). In a coordinated wave, cells beginning from a spindle morphology transition into flask cell morphology, followed by resolution to columnar morphology (Fig. 2B). As the flask cells constrict, bridges are displaced toward the narrow distal end of cells (Viamontes and Kirk 1977; Matt and Umen 2016) (Fig. 2B). A kinesin family molecular motor drives bridge displacement, which ultimately produces the force to drive inversion to completion (Nishii et al. 2003). Stresses in the connected sheet of cells induced by the cell shape changes lead to colony-wide deformation (Cole and Reedy 2003; Höhn et al. 2015). An early hypothesis that the cellular deformations eventually lead to a mechanical snap-through that passively carries the colony through the final stages of inversion (Viamontes and Kirk 1977) has been supported and refined by recent work involving a more detailed mechanical treatment (Höhn et al. 2015; Haas et al. 2018). These studies complement molecular studies that uncovered the role of a kinesin family molecular motor in displacing bridges, which ultimately provides the force to drive inversion to completion as well as that of a myosin motor in mediating by actomyosin contractility some cell shape changes required for inversion (Nishii and Ogihara 1999; Nishii et al. 2003).

Unlike multicellular sheet bending in many animals, which may involve many cellular behaviors such as growth, division, migration, and intercalation, the relative simplicity of *Volvox* inversion facilitates mathematical analysis (Höhn et al. 2015; Haas et al. 2018). As such, studies of the mechanics of *Volvox* inversion stand to clarify geometrical and mechanical aspects of cell sheet bending, which is ubiquitous in morphogenesis and movement in multicellular systems. In some cases of sheet bending, such as gastrulation (Holtfreter 1944; Odell et al. 1981; Forgács and Newman 2005; von Dassow and Davidson 2007; Rauzi et al. 2015), neurulation (Odell et al. 1981; Martin and Goldstein 2014; Vijayraghavan and Davidson 2017), placode formation (Huang et al. 2011), and primitive streak formation (Cherdantseva and Cherdantsev 2006) in various animals, polarized cell shape changes analogous to those in *Volvox* also drive sheet bending. Therefore, mechanistic studies of the physics of *Volvox* embryo inversion stand to illuminate morphogenetic processes involving cell sheet bending more generally.

Another protist system that has yielded insights into morphogenetic processes is the social amoeba *Dictyostelium*. Under starvation conditions, the normally solitary amoebae aggregate by chemotaxis to form a multicellular structure called a slug (Hagan and Cohen 1981; Weijer 2004; Fig. 2Di–iii). Slugs contain up to hundreds of thousands of individual cells and undergo coordinated, light- and temperature-directed motility to search for a suitable soil surface where the slug transforms into a stalked fruiting body that ultimately produces and releases spores (Weijer 2004; Abedin and King 2010; Fig. 2Div). Fruiting body morphogenesis arises from cellular behaviors such as directed motility, shape change, and intercalation in conjunction with physical mechanisms (Weijer 2004). The entire morphogenetic cycle, from aggregation to fruiting body, unfolds in a self-organized manner in which cells secrete and take in diffusible signals and subsequently modulate their movement and shape as well as adhesion to one another in order to undergo coordinated movement and morphogenesis (Kessler and Levine 1993; Höfer et al. 1995b; Weijer 2004).

In terms of physical mechanisms, reaction-diffusion based pattern formation is key to directing *Dictyostelium* aggregation and morphogenesis. Turing pattern formation, arising from reaction-diffusion systems, represents one of the most influential theories of physical mechanism in biology (Turing 1952; Howard et al. 2011). Pattern formation in reaction-diffusion systems stems from the dynamic interaction between local excitation, in which chemical reactions transform substances into one another, and lateral inhibition, due to diffusive transport. This competition between local activation (positive feedback) and long-range inhibition (negative feedback) is the essential feature of reaction-diffusion pattern formation (Meinhardt and Gierer 2000). The resulting patterns of chemical concentrations can take a variety of forms, from spots and stripes to spirals and traveling waves. In *Dictyostelium*, periodic cyclic adenosine monophosphate (cAMP) signals resulting from the cellular production, secretion, sensing, and degradation of cAMP drive the cellular behavior underlying aggregation as well as spatial patterning and morphogenesis at later steps of the starvation response (Shaffer 1975; Weijer 2004) (Fig. 2D). These physico-chemical patterns (Fig. 2D) orchestrate the directed crawling of cells (Fig. 2Di, ii). Specifically, cAMP reaction-diffusion patterns generally take the form of traveling linear and spiral waves (Durston 1973; Shaffer 1975; Nagano 1998; Weijer 2004). These patterns are robust to differences in cell numbers and variability in the spatial distribution of cells (Weijer 2004). They play an important role in organizing the timing and spatial organization of crawling, adhesion, and cell shape change that constitute slug formation and motility (Fig. 2Diii) and the differentiation, adhesion, secretion of ECM, cell shape change, and migration that constitute fruiting body formation (Kessler and Levine 1993; Höfer et al. 1995a; Marée and Hogeweg 2001; Weijer 2004).

Outside of *Dictyostelium*, reaction-diffusion systems have long been proposed to underlie morphogenetic processes, particularly Turing pattern formation in animal development such as in vertebrate skin patterning and limb development (Turing 1952; Oster et al. 1983). Only recently, however, has integration of experiment and theory clarified the role of cellular behaviors (such as motility and contraction) in the context of the mechanics of cells and tissues. Turing-like patterns in vertebrate skin are perhaps the best-established instances. Work in various fishes has shown that horizontal stripes of different colors stemming from different cell populations form according to a reaction-diffusion mechanism (Kondo and Asai 1995; Yamaguchi et al. 2007). Other examples of such pattern formation include hair follicle patterning in mice (Sick et al. 2006) and feather bud formation in chickens (Jung et al. 1998; Shyer et al. 2017). Studies of tooth development have also demonstrated a similar convergence of biological and physical mechanisms. In this case, morphogenesis stems from Turing-like mechanisms as well as differential tissue growth, an additional physical mechanism which drives the bending of tissues (Salazar-Ciudad and Jernvall 2002, 2010).

The existence of a similar physical mechanisms in disparate developmental contexts speaks to the generality of reaction-diffusion-based pattern formation. *Dictyostelium* represents a system of intermediate complexity between non-living reaction-diffusion systems such as the Belousov-Zhabotinsky reaction (Zhabotinsky 1964; Tyson 1979; Hudson and Mankin 1981; Petrov et al. 1993) and animals (Newman et al. 2006). The relative simplicity and tractability of *Dictyostelium*, in addition to shared physical mechanisms, presents the opportunity for insight into reaction-diffusion based coordination of cellular behavior in morphogenesis.

### Motility and navigation

Due to their small sizes and fluid environments, nearly all cells are subject to the effects of a low Reynolds number local environment, where viscous forces dominate over inertial forces (Purcell 1977). This highly viscous environment imposes constraints on motility and navigation. Unlike when we humans swim, where inertia allows us to glide for rather long distances, due to its extremely viscous environment, a cell's movement will stop within a distance equivalent to about the width of an atom if it ceases swimming. The highly viscous fluid environment of the cell also means that at the scale of a cell, there is little mixing due to turbulence. Instead, diffusion often dominates in terms of passive dispersal However, molecular transport by flow can be more important relative to diffusion for large protists (Goldstein 2015), and turbulence and other large scale flows can have marked effects on the population level spatial distribution of swimming microbes (Guasto et al. 2012).

Many protists execute directed swimming, and while the underlying mechanistic details vary, all such modalities of directed swimming are robust to the influence of diffusion, which acts to spread out chemical signals and divert microswimmers from otherwise linear trajectories. As far as protists go, the biflagellate green alga *Chlamydomonas*, a member of the order Volvocales like *Volvox*, has been extensively studied in terms of swimming behavior (Fig. 2E). Interestingly, *Chlamydomonas* can swim with two phases analogous to those of the model bacterium *Escherichia coli*: one where flagella are synchronized leading to linear trajectories and the other where flagella beat asynchronously (Fig. 2E), leading to an abrupt change in direction (Polin et al. 2009). This behavior allows *Chlamydomonas* cells to diffuse rapidly in the dark. Differential flagellar response also underlies phototaxis. Photosensing by an eyespot composed of photoreceptor proteins and pigment granules, differential flagellar beat amplitude, and asymmetric yet synchronous flagellar beating together enable directed swimming toward light (Polin et al. 2009; Bennett and Golestanian 2015; Leptos et al. 2023). Proper phototaxis relies on differential responses between the *trans-* and *cis-*flagellum (the flagellum distal and proximal to the eyespot respectively; Smyth and Berg 1982; Isogai et al. 2000). Changes in flagellar beat patterns are driven by activity of dynein molecular motors, which can be brought about by changes in cytoplasmic Ca^2+^ levels (Kamiya et al. 1984; King and Dutcher 1997; Sineshchekov and Govorunova 1999). *Chlamydomonas* rotates as it swims, and by modulating the relative beating amplitudes of its flagella, it is able to execute turns while radially scanning its environment to maintain a constant frequency of eyespot stimulation (Bennett and Golestanian 2015; Cortese and Wan 2021; Leptos et al. 2023). Navigation by organisms that rotate as they swim, ultimately following helical trajectories due to processive motion (helical klinotaxis), was first proposed as a general strategy to deterministically navigate low Reynolds environments based primarily on theoretical considerations (Crenshaw and Edelstein-Keshet 1993; Crenshaw 1993a, 1993b; Fenchel and Blackburn 1999). Subsequent work established it as an efficient and widespread means for microorganisms to locate favorable environments (Blackburn and Fenchel 1999; Fenchel and Blackburn 1999).

The mechanisms underlying phototactic swimming behavior of *Volvox* and more recently another member of the Volvocales, *Gonium*, have also been studied (Ueki et al. 2010; de Maleprade et al. 2019) but in less detail than *Chlamydomonas*. While both *Gonium* and *Volvox* are colonial, the principles by which they undergo directed swimming display interesting similarities to *Chlamydomonas*. At the cellular level, photosensory modulation of ciliary activity shows some similarities between colonial and unicellular organisms (Ueki et al. 2010; Ueki and Wakabayashi 2018). Additionally, all three organisms use rotational scanning of the environment with eyespots in conjunction with flagellar responses that are tuned to the frequency of rotation (Drescher et al. 2010; Ueki et al. 2010; Bennett and Golestanian 2015; de Maleprade et al. 2019). This synchrony allows for phototactic behavior that is robust to perturbations that would otherwise push the swimming organism off course and also to spurious changes in light due to transient environmental conditions (de Maleprade et al. 2019).

The mechanics and hydrodynamics of swimming protists have also yielded mechanistic insight. Hydrodynamic effects have also been implicated in the synchronization of flagella in various situations including motility of the green algae *Chlamydomonas* and *Volvox* (Gueron et al. 1997; Goldstein et al. 2009; Brumley et al. 2012; Friedrich and Jülicher 2012; Geyer et al. 2013; Craddock et al. 2015; Wan and Goldstein 2016). Flagellar synchrony is required for proper motility, and hydrodynamic coupling has been shown to be sufficient to produce synchrony (Gueron et al. 1997; Goldstein et al. 2009; Brumley et al. 2012; Wan and Goldstein 2016). Outside of protists, flagellar synchrony mediated by hydrodynamic interactions has been observed in spermatozoa (Machin 1963; Riedel et al. 2005; Woolley et al. 2009). In contrast to *Volvox* where hydrodynamics effects alone appear to be sufficient for coordination during swimming, flagellar synchrony in swimming *Chlamydomonas* seems to be driven by direct mechanical coupling of flagella through basal bodies as well as hydrodynamic effects and cell body movement (Geyer et al. 2013; Quaranta et al. 2015; Wan and Goldstein 2016), although the relative contributions of all of these mechanisms remains to be determined. Swimming of many ciliates is also mediated by synchronous ciliary activity (note that when referring to eukaryotes, we use the terms cilia and flagella interchangeably as they are homologous and structurally identical, and any morphological or functional differences are not relevant to our purposes here). Presumably, this may be coordinated in part through hydrodynamic effects and basal body coupling, although mechanisms of ciliary coordination in ciliates has received less attention (Párducz 1967; Machemer 1972). Although conflicting historical accounts exist, recent work in the ciliate *Euplotes*, noted for its complex locomotor behavior, has implicated mechanical cytoskeletal coupling in coordinating complex ciliary movements (Taylor 1921; Okajima and Kinosita 1966; Larson et al. 2022). Cytoskeletal elements associated with the model ciliate *Tetrahymena* have also been shown to mechanically regulate proper swimming behavior (Galati et al. 2014; Soh et al. 2020, 2022). Further, coordination and proper orientation among cilia in ciliated epithelia of animals can play an important role in human health and disease by directing the flow of mucus and other biological fluids (Chilvers et al. 2003; Mitchell et al. 2007; Crest et al. 2017; Chioccioli et al. 2019; Ramirez-San Juan et al. 2020). Again, the relative simplicity of protists presents an opportunity for detailed investigation of physical mechanisms. Such fundamental understanding with respect to physical mechanisms stands to illuminate more complex cases, for example, instances of ciliary coordination in animal epithelia, where neural control and planar cell polarity are known to be important and add additional layers of complexity (Jorissen et al. 1989; Mitchell et al. 2007; Verasztó et al. 2017; Wan 2018).

Investigating diverse forms of sensorimotor activity in protists stands to clarify general principles of cellular decision making, navigation, and motility. Combinations of a few basic sensorimotor behaviors including kinetic responses, where cells change swimming speed or direction, temporal gradient sensing, where cells sense and respond to environmental changes in scalar quantities, and helical klinotaxis (described above) have been proposed as characterizing the general strategies by which swimming protists, including diverse flagellates and ciliates, locate favorable environments (Fenchel and Blackburn 1999). Even restricted to flagellar-based swimming, the modalities detailed in the preceding paragraphs represent only a few of the many diverse modes of protist locomotion. For example, the rosette colonies (rosettes) *Salpingoeca rosetta*, a species of choanoflagellate, the closest living relatives of animals, swim along helical trajectories and undergo directed motility in response to oxygen gradients (Kirkegaard et al. 2016). Unlike directed motility in the previously described systems, in rosettes, there is no flagellar coordination. Instead, directed motility, which can be described by an aggregate random walker model, arises from the joint independent behavior of cells that modulate their flagellar beating based on changes in oxygen levels, leading to changes in colony swimming direction with a frequency dependent on oxygen concentration (Kirkegaard et al. 2016). Crawling cells can also exhibit directed motility, with *Dictyostelium* being one of the best studied systems among the amoeboid protists, which often navigate by sensing gradients over their bodies (King and Insall 2009; Wan and Jékely 2021). Work investigating *Dictyostelium* navigation in spatially complex environments using a combination of theory and experiments has demonstrated the importance of self-generated gradients, which may also play an important role in animal cell navigation in health and disease (Tweedy et al. 2016, 2020; Tweedy and Insall 2020). In nature, chemotaxis and cell motility more generally can play an important role in nutrient cycling at the ecosystem and even global scale (Guasto et al. 2012). At a population level, the interaction between active swimming and shear due to natural turbulence in aquatic environments can lead to up to a 10-fold increase in local phytoplankton density (Durham et al. 2013). Therefore, understanding these mechanisms and dynamics in their environmental context will be important for understanding nutrient cycling in myriad ecosystems where protists play important roles.

### Decision making

Spanning elements of morphogenesis as well as navigation, the slime mold *P. polycephalum* has become a model for studying how cells coordinate complex behaviors in terms of computation and decision-making (Nakagaki et al. 2000; Tero et al. 2010; Beekman and Latty 2015; Reid et al. 2016). *Physarum* is a large (easily visible to the naked eye), plasmodial, or multinucleated, cell that grows as a dynamic network of interconnected, contractile tubes through which cytoplasm is rhythmically pumped back and forth ( Matsumoto et al. 2008). In nature, *Physarum* crawls across forest environments in search of food and favorable environments, which are generally dark and damp (Fig. 2F). Upon finding food, the cell alters its structure to increase cytoplasmic flow and growth toward the food, which it then digests extracellularly or engulfs by phagocytosis depending on the food source (Goodman 1980; Bailey 1995; Matsumoto et al. 2008). Foraging proceeds in two phases: an exploratory expansion phase in which the cell grows outward, growing its network, and a contraction phase in which the cell prunes back regions that did not encounter nutrients while expanding tube diameters in regions involved in nutrient transport (Nakagaki et al. 2001). Extracellular secretions may serve as a self-produced cue for *Physarum* to avoid exploring regions it has already visited (Reid et al. 2012, 2013). In the lab, *Physarum* has been shown to be able to solve mazes (Nakagaki et al. 2000), to find the shortest distance between points of interest (Tero et al. 2010), a notoriously difficult computational problem, and to organize its structure to optimize its diet (Dussutour et al. 2010). *Physarum* also displays strategic decision making when confronted with conflicting environmental stimuli. When presented with a choice between high and low quality food in different light conditions (with light generally evoking an aversive response), cells tend to choose the high-quality food regardless of light condition once the difference in quality between the two food sources is high enough (Latty and Beekman 2009). The modulation of foraging behavior based on risk (light) exposure shows that *Physarum* can make multi-objective foraging decisions. Together, these examples illustrate *Physarum*’s capacity for making the kinds of complex decisions often associated with nervous systems.

In order to accomplish this kind of sophisticated behavior, the undifferentiated cell needs to coordinate morphogenesis across centimeters. Diffusion alone would be prohibitively slow, taking even ions days to travel such distances assuming a cell size of 2 cm and diffusion on the order of 10^−5^ m^2^/s. However, cytoplasmic flow driven by rhythmic actomyosin mediated contractions of the gel-like tubular cell structure can transport signals much more rapidly (Matsumoto et al. 2008; Alim et al. 2013). Flow increases Taylor dispersion, the effective dispersion of molecular substances beyond pure diffusivity, and work has shown that *Physarum* can reorganize its structure to optimize Taylor dispersion (Marbach et al. 2016; Alim et al. 2017). Additionally, behavioral coordination arises in a self-organizing manner as the cell lacks any sort of centralized organizational structure (Tsuda et al. 2004; Tero et al. 2010; Alim et al. 2013, 2017; Beekman and Latty 2015; Reid et al. 2016). Quantitative analysis of *Physarum* dynamics has shown how advected cues, likely intracellular Ca^2+^ (Alim et al. 2017), along with changes in network structure and tube diameter affected by those signals, are sufficient to account for the observed foraging behaviors (Matsumoto et al. 1986; Tero et al. 2010; Alim et al. 2013, 2017). In this way, the cell is an active reaction diffusion system (Nakagaki et al. 1999; Yamada et al. 1999; Adamatzky 2007).

In *Physaurm*, analysis of biophysical processes has provided mechanistic insight into the coordination of complex behaviors. It has also allowed for the testing of competing hypotheses as to the underlying molecular mechanisms of signal propagation (i.e., elastic waves vs. electrical impulses vs. advected molecular signals) (Alim et al. 2017). Insights from this work may inform our understanding of systems beyond *Physarum* including hyphal networks by which fungi grow and feed, which share many physical similarities. More generally, biophysical approaches to analyzing decision making can also inform our understanding of how cells and other “simple” systems are able to coordinate and control complex behavior and of how living systems perform computational tasks (Beekman and Latty 2015; Reid et al. 2016; Alim et al. 2017; Oettmeier et al. 2017; Coyle et al. 2019; Larson et al. 2022).

After a time of considerable interest in the early to mid 20th century, ciliates have re-emerged as a system for studying decision making and even learning in single cells (Jennings 1906; Bennet and Francis 1972; Coyle et al. 2019; Dexter et al. 2019; Marshall 2019; Trinh et al. 2019; Gershman et al. 2021; Larson et al. 2022; Rajan et al. 2023). Ciliates are attractive systems for studying cellular decision making owing in part to their obvious, rapid, stereotyped behaviors, along with their polarized and highly stereotyped cell morphologies that together make them amenable to rigorous analyses. Cytoskeletal mechanics have been implicated in coordinating complex movement patterns of *Lacrymaria*, a predator noted for its long, flexible neck used for hunting and of *Euplotes*, a cell that walks using leg-like bundles of cilia (Coyle et al. 2019; Larson et al. 2022). Experiments in *Stentor*, a cell perhaps best known for its impressive capacity for regeneration, grounded in quantitative analysis in concert with mathematical modeling have demonstrated sequential logic in avoidance reactions in response to aversive stimuli as well as discrete cell state changes involved in learning to ignore a benign mechanical stimulus (Dexter et al. 2019; Rajan et al. 2023). Similarly to *Stentor, Physarum* is capable of habituation-based learning, in which cells can learn to ignore aversive chemical stimuli (Boisseau et al. 2016). Fascinatingly, conditioned responses in *Physaruym* can be transferred by cell fusion (Vogel and Dussutour 2016; Boussard et al. 2018). Generally, the mechanistic underpinnings of these complex computational processes in cells remain to be clarified, although sodium may function as a substrate for encoding memories during *Physarum* learning (Boussard et al. 2018). Extending insights from decision making in ciliates to other eukaryotes stands to advance understanding of similar mechanisms and processes that underlie cellular function. Recently, work combining theory and experiments to model sensorimotor behavior in *Paramecium*, a longstanding model ciliate, has initiated such an approach, comparing the cell with its calcium-based action potential triggered movements to a swimming neuron (Brette 2021; Elices et al. 2023).

## Evolution at the intersection of biology and physics

While considerable work on the intersection of physical constraints and cellular behavior has identified mechanisms of organismal function, less work has focused on the role of this coaction in evolution, leaving mostly open a frontier at the interface of biology and physics. There is, however, precedent for such studies, although most work has focused on animals and plants at or above the tissue scale, without explicit treatment of cells, and has tended to emphasize phenotypic limitations and optimization of function, yielding a rather narrow view of the role of physical constraints and mechanism.

For example, work on the evolution of ammonite shell coiling grounded in a theoretical morphospace (Raup 1967) explained the lack of certain shell morphologies (Chamberlain and Westermann 1976) and predicted the existence of others, which were later discovered (Chamberlain 1981), based on hydrodynamic considerations of swimming efficiency (Raup 1967; Chamberlain and Westermann 1976). Another prominent example comes from the investigation of the evolution of plant morphologies. Niklas and Kerchner developed a simulation framework using a rule-based model of plant growth grounded in physical and physiological constraints was able to capture much of the evolutionary trajectory of land plants (Niklas and Kerchner 1984; Niklas 1988, 1997, 1999, 2013). This simulation framework was also able to generate specific predictions about how the intensity and diversity of selective pressures (the roughness of the fitness landscape) along with developmental constraints affect the evolution of morphological diversity and complexity (Niklas 1999). While the previous two examples do not explicitly treat the behavior of cells underlying morphogenesis, evolution at the intersection of cellular behavior and physical constraints has also been considered. The theory of Turing patterns involving behaviors such as migration and haptotaxis, in addition to mechanics (Newman and Frisch 1979; Oster et al. 1983, 1988; Murray and Oster 1984; Murray et al. 1988), has recently been tested and built upon in the context of vertebrate limb (Sheth et al. 2012; Cooper et al. 2014; Lopez-Rios et al. 2014; Cooper 2015; Newman et al. 2018) and tooth evolution and development (Salazar-Ciudad and Jernvall 2002; Salazar-Ciudad 2012). Results from these studies show how even in complex biological processes, the underlying control parameters may not be so numerous (Salazar-Ciudad 2012; Newman et al. 2018). While not focused on protists, research on morphogenesis in plants and animals highlights how considering physical mechanisms can sharpen focus on essential molecular or genetic components.

Investigation of physical mechanisms naturally fits into an evolutionary context because biophysical models can generate predictions about the relationships between changes in cellular properties or behaviors and resultant phenotypes. Studies combining biology and physics to understand protist evolution are less extensive compared to those described so far. These studies have also tended to focus primarily on adaptation and optimal performance. Work on the evolution of volvocine algae in the context of physical constraints has perhaps received the most attention (Goldstein 2015). Experiments and theory together suggest important physical constraints on colony size scaling. First, experiments showed that external fluid flow is important for *Volvox* colony metabolism. Specifically, germ cells of deflagellated colonies did not grow well, but growth could be rescued by increasing external flow by stirring (Solari et al. 2006). Experiments and theory then demonstrated that *Volvox* colonies were able to maintain nutrient flux through phenotypic plasticity (increasing cell spacing and flagellar length) even in the face of reduced nutrient growth media (Solari et al. 2011), while the unicellular relative *Chlamydomonas* did not show such compensatory phenotypic plasticity (Solari et al. 2011). Theory suggested that for large spherical colonies, transport of nutrients by flagellar driven flow should play an important role (Short et al. 2006; Goldstein 2015). This is because nutrient uptake scales proportionally with radius while metabolic needs of the colony will scale as the square of the radius (Short et al. 2006; Goldstein 2015), so metabolic needs outpace the increase in diffusive transport of nutrients with increasing colony size. Increasing swimming speed, however can circumvent this constraint by increasing nutrient transport (Short et al. 2006; Goldstein 2015). These results and arguments suggest that, independently of other selective pressures and constraints, large colonies should optimize flow around colonies via swimming to maximize growth rates. Interestingly, the predicted point of decreasing diffusive uptake with colony size sits near the middle of the size of *Volvox* colonies (Short et al. 2006; Goldstein 2015).

Theory and experiments have also considered optimality of protist morphology for swimming in *Chlamydomonas* (Tam and Hosoi 2011; Bauer et al. 2021) and feeding in choanoflagellates (Roper et al. 2013; Kirkegaard and Goldstein 2016; Asadzadeh et al. 2019). Studies of *Chlamydomonas* found that flagellar lengths on average sit near the optimal length for swimming and gliding speeds (Bauer et al. 2021) and that the modes of swimming employed by *Chlamydomonas* are themselves close to optimal for the size and shape of the organism (Kuchka and Jarvik 1987; Tam and Hosoi 2011; Khona et al. 2013). Work on choanoflagellates has argued that colony morphology may be optimized for cooperative feeding (Roper et al. 2013). However, this result is contentious, with theoretical work arguing that different environmental tradeoffs exist for different life history stages (Kirkegaard and Goldstein 2016). Computational fluid mechanics modeling was also used to test hypotheses about the function of lorica, silica cages produced by some choanoflagellates (Asadzadeh et al. 2019). Simulations, calibrated by data, showed that the lorica does not enhance feeding by increasing drag and preventing feeding current recirculation as previously hypothesized (Leadbeater 2015; Asadzadeh et al. 2019), but instead may increase prey capture efficiency by stabilizing cellular motion (Asadzadeh et al. 2019).

In addition to explaining limitations on evolutionary outcomes, investigating the interplay between active processes, and physical constraints can also bring deeper or more general understanding of mechanism, both in terms of conserved and convergent processes. In particular, the generality of the physical context of many evolutionary processes can lead to the emergence of rules in evolutionary dynamics. Experimental evolution constitutes a powerful method by which to understand these rules. Investigations of bacterial evolution have tended to dominate work in experimental evolution. The reproducibly, convergently evolved “wrinkly spreader” phenotype in *Pseudomonas fluorescens*, characterized by a wrinkled surface to increase surface area for oxygen absorption, provides a salient example of phenotypic convergence in the face of physical constraints (Spiers et al. 2002; Spiers 2014; Lind et al. 2015, 2017). However, eukaryotic systems including protists stand to provide insights as well. Yeast and *Sphaeroforma*, a close relative of animals, have already illuminated the evolution of multicellularity along these lines, demonstrating the potential of such approaches (Libby et al. 2014; Jacobeen et al. 2018; Day et al. 2022; Dudin et al. 2022). Recent work has argued that constraints imposed by cell packing may be unavoidable in the evolution of simple multicellularity (Jacobeen et al. 2018; Day et al. 2022). In the experimental evolution of snowflake yeast, a class of lab evolved strains of multicellular *Saccharomyces cerevisiae* that form by incomplete cytokinesis, under selection for larger colony size, cells reproducibly evolve to become more elongated in order to reduce accumulated stress that would otherwise fragment colonies at larger colony sizes (Jacobeen et al. 2018). The authors argue that accumulated stress may in fact be a generic feature of developmental, multicellular systems (Jacobeen et al. 2018). In this case, accumulated residual stress acts as both a constraint (by limiting overall colony size) and a mechanism of multicellular reproduction by causing fragmentation of colonies. Another study of experimental evolution of snowflake yeast in conjunction with mathematical modeling argued that accounting for cell packing geometry was key to understanding the counterintuitive evolution of increasing rates of apoptosis (Libby et al. 2014). Recently, work showed that changes in cell morphology leading to dense entanglement of cells were key to overcoming colony fragmentation due to accumulated stress in the evolution of macroscopic snowflake yeast (Bozdag et al. 2023). In these studies, geometrical constraints proved to play a key role in explaining evolutionary processes. Similar constraints on cell packing have been implicated in the evolution of diverse colony morphologies among choanoflagellates (Larson et al. 2020).

These examples, though not all dealing with protists, illustrate how understanding of physical constraints can elucidate rules in evolution. Furthermore, in several cases, physical constraints played a key role in clarifying the functional or adaptive significance of cellular modification. The broad spectrum of cell biology represented by microbial eukaryotes along with the development of new model systems points to the potential for a great expansion of research in this direction. Experimental evolution using protists would greatly benefit from high quality genomes and defined culture conditions in addition to high throughput methods for quantifying diverse phenotypes.

## Conclusion

Together, the work reviewed here illustrates the power in combining perspectives from physics and biology to understand principles of function and evolution in biological systems. Importantly, it shows how investigating biological function in terms of the regulated interplay between cellular behavior and physical constraints drives mechanistic insight. Additionally, the reviewed examples serve to illustrate the value of close collaboration between theory and experiment. As we have seen, physical constraints can act not only as limitations but can also underpin robust mechanisms and can highlight shared principles among diverse organisms spanning the tree of life.

In general, protists present a rich, comparatively unexplored region of cell biology ripe for investigation by quantitative methods. The examples presented here have barely scratched the surface of protist diversity. We are only beginning to understand the principles of cell behavior and how they play out beyond the most intensively studied systems. In addition to seeking out new biological insight in the lab or in the field, the historical cell biology literature represents an extensive resource for interesting observations and questions that can now be effectively addressed with modern methods. Advances in microscopy and computation along with molecular techniques stand to continue to drive deep mechanistic insight into fundamental processes across the stunning diversity of eukaryotic cells. Promising future directions include investigating how cellular behaviors are controlled and play out in natural or naturalistic environmental contexts, the continued development of phylogenetically diverse model systems including cultivation methods and natural history studies (Keeling 2019) as well as the discovery and isolation of new organisms from the field, using comparative approaches to study the evolution of cellular behavior, and capitalizing on recent progress in the physics of behavior to quantitatively characterize the behavioral repertoires of cells (Bialek 2022). At the intersection of these lines of research lie the following interrelated questions: How are behaviorally relevant geometrical features of cells such as the size, shape, number, position of appendages or other structures controlled, and how do these features afford behaviors? How do cells manage the flow of information across their bodies to properly execute behaviors in specific environmental contexts? Continued synthesis of complementary perspectives on diverse systems will clarify unifying themes and fundamental biological principles.

## Notes

From the symposium “Large-scale biological phenomena arising from small-scale biophysical processes” presented at the virtual annual meeting of the Society for Integrative and Comparative Biology, January 16–March 31, 2023.

## References

[bib1] Abedin M , King N 2010. Diverse evolutionary paths to cell adhesion. Trends in Cell Biology. 20:734–42. 20817460 10.1016/j.tcb.2010.08.002 PMC2991404

[bib2] Adamatzky A 2007. Physarum machines: encapsulating Reaction–diffusion to compute spanning tree. Naturwissenschaften. 94:975–80. 17603779 10.1007/s00114-007-0276-5

[bib3] Alim K , Amselem G, Peaudecerf F, Brenner MP, Pringle A 2013. Random network peristalsis in *Physarum polycephalum* organizes fluid flows across an individual. Proc Natl Acad Sci USA. 110:13306–11. 23898203 10.1073/pnas.1305049110 PMC3746869

[bib4] Alim K , Andrew N, Pringle A, Brenner MP 2017. Mechanism of signal propagation in *Physarum polycephalum*. Proc Natl Acad Sci USA. 114:5136–41. 28465441 10.1073/pnas.1618114114 PMC5441820

[bib5] Asadzadeh SS , Nielsen LT, Andersen A, Dölger J, Kiørboe T, Larsen PS, Walther JH 2019. Hydrodynamic functionality of the lorica in choanoflagellates. J R Soc Interface. 16:20180478. 30958164 10.1098/rsif.2018.0478 PMC6364640

[bib6] Ashby WR 1956. An Introduction to Cybernetics London. London, UK: Chapman & Hall.

[bib7] Bailey J 1995. Plasmodium development in the myxomycete physarum polycephalum: genetic Control and cellular events. Microbiology. 141:2355–65. 7581996 10.1099/13500872-141-10-2355

[bib8] Baldauf SL , Palmer JD, Doolittle WF 1996. The root of the universal tree and the origin of eukaryotes based on elongation factor phylogeny. Proc Natl Acad Sci USA. 93:7749–54. 8755547 10.1073/pnas.93.15.7749 PMC38819

[bib9] Bauer D , Ishikawa H, Wemmer KA, Hendel NL, Kondev J, Marshall WF 2021. Analysis of biological noise in the flagellar length control system. Iscience. 24:102354. 33898946 10.1016/j.isci.2021.102354 PMC8059064

[bib10] Beekman M , Latty T 2015. Brainless but multi-headed: decision making by the acellular slime mould *physarum polycephalum*. J Mol Biol. 427:3734–43. 26189159 10.1016/j.jmb.2015.07.007

[bib11] Bennet DA , Francis D 1972. Learning in stentor. J Protozool. 19:484–7.

[bib12] Bennett RR , Golestanian R 2015. A steering mechanism for phototaxis in Chlamydomonas. J R Soc Interface. 12:104. 10.1098/rsif.2014.1164 PMC4345482 25589576

[bib13] Bialek W 2022. On the dimensionality of behavior. Proc Natl Acad Sci USA. 119:e2021860119. 35486689 10.1073/pnas.2021860119 PMC9170048

[bib14] Blackburn N , Fenchel T 1999. Modelling of microscale patch encounter by Chemotactic Protozoa. Protist. 150:337–43. 10575705 10.1016/S1434-4610(99)70034-9

[bib15] Boisseau RP , Vogel D, Dussutour A 2016. Habituation in non-neural organisms: evidence from slime moulds. Proc R Soc B Biol Sci. 283:20160446. 10.1098/rspb.2016.0446 PMC4855389 27122563

[bib16] Boussard A , Delescluse J, Pérez-Escudero A, Dussutour A 2018. Memory inception and preservation in slime moulds: the quest for a common mechanism. Philos Trans R Soc B Biol Sci. 374:20180368. 10.1098/rstb.2018.0368 PMC6553583 31006372

[bib17] Bozdag GO , Zamani-Dahaj SA, Day TC, Kahn PC, Burnetti AJ, Lac DT, Tong K, Conlin PL, Balwani AH, Dyer EL et al. 2023. De novo evolution of macroscopic multicellularity. Nature. 617:747–54. 37165189 10.1038/s41586-023-06052-1 PMC10425966

[bib18] Brette R 2021. Integrative Neuroscience of Paramecium, a “swimming neuron.” Eneuro. 8. 10.1523/ENEURO.0018-21.2021 PMC8208649 33952615

[bib19] Brumley DR , Polin M, Pedley TJ, Goldstein RE 2012. Hydrodynamic synchronization and metachronal waves on the surface of the colonial Alga *Volvox carteri*. Phys Rev Lett. 109:268102. 23368623 10.1103/PhysRevLett.109.268102

[bib20] Brunet T , King N 2017. The origin of animal multicellularity and cell differentiation. Dev Cell. 43:124–40. 29065305 10.1016/j.devcel.2017.09.016 PMC6089241

[bib21] Burton K , Taylor DL 1997. Traction forces of cytokinesis measured with optically modified elastic substrata. Nature. 385:450–4. 9009194 10.1038/385450a0

[bib22] Calkins GN 1926. The biology of Protozoa. Philadelphia, PA: Lea & Febiger. 10.5962/bhl.title.7233

[bib23] Cavalier-Smith T 1997. Amoeboflagellates and mitochondrial cristae in eukaryote evolution: megasystematics Of the new protozoan subkingdoms eozoa and neozoa. Arch Fur Protistenkd. 143:237–258.

[bib24] Chamberlain JA , Westermann GEG 1976. Hydrodynamic properties of cephalopod shell ornament. Paleobiology. 2:316–31.

[bib25] Chamberlain JAJ 1981. Hydromechanical design of fossil cephalopods. Syst Assoc Spec Ed Ammonoidea. 18:289–336.

[bib26] Cherdantseva EM , Cherdantsev VG 2006. Geometry and mechanics of teleost gastrulation and the formation of primary embryonic axes. Int J Dev Biol. 50:157–68. 16479485 10.1387/ijdb.052059ec

[bib27] Chilvers MA , Rutman A, O'callaghan C 2003. Functional analysis of cilia and ciliated epithelial ultrastructure in healthy children and young adults. Airw Biol Thorax. 58:333–8. 10.1136/thorax.58.4.333 PMC1746630 12668798

[bib28] Chioccioli M , Feriani L, Kotar J, Bratcher PE, Cicuta P 2019. Phenotyping ciliary dynamics and coordination in response to CFTR-modulators in Cystic Fibrosis respiratory epithelial cells. Nat Commun. 10:1763. 30992452 10.1038/s41467-019-09798-3 PMC6467870

[bib29] Cole DG , Reedy MV 2003. Algal morphogenesis: how volvox turns itself inside-out. Curr Biol. 13:R770–R772. 14521856 10.1016/j.cub.2003.09.019

[bib30] Collier JL , Rest JS 2019. Swimming, gliding, and rolling toward the mainstream: cell Biology of marine protists. MBoC. 30:1245–8. 31084566 10.1091/mbc.E18-11-0724 PMC6724603

[bib31] Cooper KL 2015. Self-organization in the limb: a turing Mechanism for digit development. Curr Opin Genet Dev. 32:92–7. 25819977 10.1016/j.gde.2015.02.001

[bib32] Cooper KL , Sears KE, Uygur A, Maier J, Baczkowski K-S, Brosnahan M, Antczak D, Skidmore JA, Tabin CJ 2014. Patterning and post-patterning modes of evolutionary digit loss in mammals. Nature. 511:41–5. 24990742 10.1038/nature13496 PMC4228958

[bib33] Cortese D , Wan KY 2021. Control of helical navigation by three-dimensional flagellar beating. Phys Rev Lett. 126:088003. 33709750 10.1103/PhysRevLett.126.088003 PMC7617974

[bib34] Coyle SM , Flaum EM, Li H, Krishnamurthy D, Prakash M 2019. Coupled active systems encode an emergent hunting behavior in the unicellular predator *Lacrymaria olor*. Curr Biol. 29:3838–3850.e3. 31679941 10.1016/j.cub.2019.09.034 PMC7511173

[bib35] Craddock TJA , Hameroff SR, Ayoub T.A, Klobukowski M, Tuszynski JA. 2015. Anesthetics act in quantum channels in brain microtubules to prevent consciousness. CTMC. 15:523–33. 10.2174/1568026615666150225104543 25714379

[bib36] Crenshaw H 1993. Orientation by helical motion—I. Kinematics of the helical motion of organisms with up to six degrees of freedom. Bltn Mathcal Biology. 55:197–212.

[bib37] Crenshaw H 1993. Orientation by helical motion—III. Microorganisms can orient to stimuli by changing the direction of their rotational velocity. Bltn Mathcal Biology. 55:231–55.

[bib38] Crenshaw HC , Edelstein-Keshet L 1993. Orientation by helical motion-II. Changing the direction of the axis of motion. Bltn Mathcal Biology. 55:213–30.

[bib39] Crest J , Diz-Muñoz A, Chen D-Y, Fletcher DA, Bilder D 2017. Organ sculpting by patterned extracellular matrix stiffness. Elife. 6:24958. 10.7554/eLife.24958 PMC5503509 28653906

[bib40] Day TC , Höhn SS, Zamani-Dahaj SA, Yanni D, Burnetti A, Pentz J, Honerkamp-Smith AR, Wioland H, Sleath HR, Ratcliff WC et al. 2022. Cellular organization in lab-evolved and extant multicellular species obeys a maximum entropy law. Elife. 11:72707. 10.7554/eLife.72707 PMC8860445 35188101

[bib41] de Maleprade H , Moisy F, Ishikawa T, Goldstein RE 2019. Motility and Phototaxis of Gonium, the Simplest Differentiated Colonial Alga. Phys Rev E. 101:002416. 10.1103/PhysRevE.101.022416 PMC7616084 32168596

[bib42] Dexter JP , Prabakaran S, Gunawardena J 2019. A complex hierarchy of avoidance behaviors in a single-cell eukaryote. Curr Biol. 29:4323–4329.e2. 31813604 10.1016/j.cub.2019.10.059

[bib43] Dormann D , Vasiev B, Weijer C 2002. Becoming multicellular by aggregation; the morphogenesis of the social amoebae *dicyostelium discoideum*. J Biol Phys. 28:765–80. 23345812 10.1023/A:1021259326918 PMC3456464

[bib44] Dorn JF , Zhang L, Phi T-T, Lacroix B, Maddox PS, Liu J, Maddox AS 2016. A theoretical model of cytokinesis implicates feedback between membrane curvature and cytoskeletal organization in asymmetric cytokinetic furrowing. MBoC. 27:1286–99. 26912796 10.1091/mbc.E15-06-0374 PMC4831882

[bib45] Drescher K , Goldstein RE, Tuval I 2010. Fidelity of adaptive phototaxis. Proc Natl Acad Sci USA. 107:11171–6. 20534560 10.1073/pnas.1000901107 PMC2895142

[bib46] Dröscher A 2014. History of cell biology. In: Encyclopedia of Life Sciences, Chichester, UK: John Wiley & Sons, Ltd.

[bib47] Dudin O , Wielgoss S, New AM, Ruiz-Trillo I 2022. Regulation of sedimentation rate shapes the evolution of multicellularity in a close unicellular relative of animals. PLOS Biol. 20:e3001551. 35349578 10.1371/journal.pbio.3001551 PMC8963540

[bib48] Durham WM , Climent E, Barry M, De Lillo F, Boffetta G, Cencini M, Stocker R 2013. Turbulence drives microscale patches of motile phytoplankton. Nat Commun. 4:2148 23852011 10.1038/ncomms3148

[bib49] Durston AJ 1973. Dictyostelium discoideum aggregation fields as excitable media. J Theor Biol. 42:483–504. 4358315 10.1016/0022-5193(73)90242-7

[bib50] Dussutour A , Latty T, Beekman M, Simpson SJ 2010. Amoeboid organism solves complex nutritional challenges. Proc Natl Acad Sci USA. 107:4607–11. 20142479 10.1073/pnas.0912198107 PMC2842061

[bib51] Elde NC , Morgan G, Winey M, Sperling L, Turkewitz AP 2005. Elucidation of clathrin-mediated endocytosis in tetrahymena reveals an evolutionarily convergent recruitment of dynamin. PLoS Genet. 1:e52. 16276403 10.1371/journal.pgen.0010052 PMC1277907

[bib52] Elices I , Kulkarni A, Escoubet N, Pontani LL, Prevost AM, Brette R 2023. An electrophysiological and kinematic model of paramecium, the “swimming neuron.” PLOS Comput Biol. 19:e1010899. 36758112 10.1371/journal.pcbi.1010899 PMC9946239

[bib53] Faktorová D , Nisbet RER, Fernández Robledo JA, Casacuberta E, Sudek L, Allen AE, Ares M, Aresté C, Balestreri C, Barbrook AC et al. 2020. Genetic tool development in marine protists: emerging Model organisms for experimental cell biology. Nat Methods. 17:1–14. 32251396 10.1038/s41592-020-0796-x PMC7200600

[bib54] Fenchel T , Blackburn N 1999. Motile chemosensory behaviour of phagotrophic protists: mechanisms for and efficiency in congregating at food patches. Protist. 150:325–36. 10575704 10.1016/S1434-4610(99)70033-7

[bib55] Forgács G , Newman SA 2005. Biological Physics of the Developing Embryo. Cambridge, UK: Cambridge University Press.

[bib56] Friedrich BM , Jülicher F 2012. Flagellar synchronization independent of hydrodynamic interactions. Phys. Rev. Lett. 109:138102. 23030122 10.1103/PhysRevLett.109.138102

[bib57] Fritz-Laylin LK , Prochnik SE, Ginger ML, Dacks JB, Carpenter ML, Field MC, Kuo A, Paredez A, Chapman J, Pham J et al. 2010. The genome of Naegleria gruberi illuminates early eukaryotic versatility. Cell. 140:631–42. 20211133 10.1016/j.cell.2010.01.032

[bib58] Galati DF , Bonney S, Kronenberg Z, Clarissa C, Yandell M, Elde NC, Jerka-Dziadosz M, Giddings TH, Frankel J, Pearson CG 2014. DisAp-dependent striated fiber elongation is required to organize ciliary arrays. J Cell Biol. 207:705–15. 25533842 10.1083/jcb.201409123 PMC4274257

[bib59] Gershman SJ , Balbi PE, Gallistel CR, Gunawardena J 2021. Reconsidering the evidence for learning in single cells. Elife. 10:61907. 10.7554/eLife.61907 PMC7781593 33395388

[bib60] Geyer VF , Jülicher F, Howard J, Friedrich BM 2013. Cell-body rocking is a dominant mechanism for flagellar synchronization in a swimming alga. Proc Natl Acad Sci USA. 110:18058–63. 24145440 10.1073/pnas.1300895110 PMC3831503

[bib61] Goldstein RE 2015. Green algae as model organisms for biological fluid dynamics. Annu Rev Fluid Mech. 47:343–75. 26594068 10.1146/annurev-fluid-010313-141426 PMC4650200

[bib62] Goldstein RE , Polin M, Tuval I 2009. Noise and synchronization in pairs of beating *eukaryotic flagella*. Phys Rev Lett. 103:168103. 19905728 10.1103/PhysRevLett.103.168103

[bib63] Goodman EM 1980. Physarum polycephalum: a review of a model system using a structure-function approach. Int Rev Cytol. 63:1–58.

[bib64] Gould SB , Waller RF, McFadden GI 2008. Plastid Evolution. Annu Rev Plant Biol. 59:491–517. 18315522 10.1146/annurev.arplant.59.032607.092915

[bib65] Gray MW 1989. The evolutionary origins of organelles. Trends Genet. 5:294–9. 2686121 10.1016/0168-9525(89)90111-x

[bib66] Guasto JS , Rusconi R, Stocker R 2012. Fluid mechanics of planktonic microorganisms. Annu Rev Fluid Mech. 44:373–400.

[bib67] Guermazi W , Elloumi J, Ayadi H, Bouain A, Aleya L 2008. Rearing of Fabrea salina Henneguy (Ciliophora, Heterotrichida) with three unicellular feeds. Comptes Rendus Biologies. 331:56–63. 18187123 10.1016/j.crvi.2007.10.006

[bib68] Gueron S , Levit-Gurevich K, Liron N, Blum JJ, Baroud CN 1997. Cilia internal mechanism and metachronal coordination as the result of hydrodynamical coupling. Proc Natl Acad Sci USA. 94:6001–6. 9177158 10.1073/pnas.94.12.6001 PMC20990

[bib69] Haas PA , Goldstein RE 2018. Embryonic inversion in *Volvox carteri* : the flipping and peeling of elastic lips. Phys Rev E. 98:052415. 30519671 10.1103/PhysRevE.98.052415 PMC6276994

[bib70] Haas PA , Höhn SSMH, Honerkamp-Smith AR, Kirkegaard JB, Goldstein RE 2018. The noisy basis of morphogenesis: mechanisms and mechanics of cell sheet folding inferred from developmental variability. PLOS Biol. 16:e2005536. 30001335 10.1371/journal.pbio.2005536 PMC6063725

[bib71] Haeckel E 1904. Kunstformen Der Natur, Leipzig und Wien, DE: Verlag des Bibliographischen Instituts.

[bib72] Hagan PS , Cohen MS 1981. Diffusion-induced morphogenesis in the development of dictyostelium. J Theor Biol. 93:881–908.

[bib73] Hake KH , West PT, McDonald K, Laundon D, Bayonas AGDL, Feng C, Burkhardt P, Richter DJ, Banfield JF, King N. 2021. Colonial choanoflagellate isolated from Mono Lake harbors a microbiome. bioRxiv. 2021.03.30.437421. 10.1128/mbio.01623-24 PMC11389367 39140743

[bib74] Höfer T , Sherratt JA, Maini PK 1995a. Cellular pattern formation during dictyostelium aggregation. Physica D: Nonlinear Phenomena. 85:425–44.

[bib75] Höfer T , Sherratt JA, Maini PK 1995b. Dictyostelium discoideum : cellular Self-organization in an excitable biological medium. Proc R Soc London Ser B Biol Sci. 259:249–57. 10.1098/rspb.1995.0037 7740045

[bib76] Höhn S , Honerkamp-Smith AR, Haas PA, Trong PK, Goldstein RE 2015. Dynamics of a Volvox embryo turning itself inside out. Phys Rev Lett. 114:178101. 25978266 10.1103/PhysRevLett.114.178101

[bib77] Holtfreter J 1944. A study of the mechanics of gastrulation. J Exp Zool. 95:171–212.

[bib78] Howard J , Grill SW, Bois JS 2011. Turing's next steps: the Mechanochemical basis of morphogenesis. Nat Rev Mol Cell Biol. 12:392–8. 21602907 10.1038/nrm3120

[bib79] Huang J , Rajagopal R, Liu Y, Dattilo LK, Shaham O, Ashery-Padan R, Beebe DC 2011. The mechanism of lens placode formation: a Case of matrix-mediated morphogenesis. Dev Biol. 355:32–42. 21540023 10.1016/j.ydbio.2011.04.008 PMC3104088

[bib80] Hudson JL , Mankin JC 1981. Chaos in the Belousov–Zhabotinskii reaction. J Chem Phys. 74:6171–7.

[bib81] Isogai N , Kamiya R, Yoshimura K 2000. Dominance between the two flagella during phototactic turning in Chlamydomonas. Zool Science. 17:1261–6.

[bib82] Jacobeen S , Pentz JT, Graba EC, Brandys CG, Ratcliff WC, Yunker PJ 2018. Cellular packing, mechanical stress and the evolution of multicellularity. Nature Phys. 14:286–90. 31723354 10.1038/s41567-017-0002-y PMC6853058

[bib83] Jennings HS 1906. Behavior of The Lower Organisms., Behavior of The Lower Organisms. New York, NY: Columbia University Press.

[bib84] Jorissen M , Van der Schueren B, Tyberghein J, Van der Berghe H, Cassiman JJ 1989. Ciliogenesis and coordinated ciliary beating in human nasal epithelial cells cultured in vitro. Acta Otorhinolaryngol Belg. 43:67–73. 2801098

[bib85] Jung H-S , Francis-West PH, Widelitz RB, Jiang T-X, Ting-Berreth S, Tickle C, Wolpert L, Chuong C-M 1998. Local inhibitory action of bmps and their relationships with activators in feather formation: implications for periodic patterning. Dev Biology. 196:11–23. 10.1006/dbio.1998.8850 9527877

[bib86] Kamiya R , 1984. Submicromolar levels of calcium control the balance of beating between the two flagella in demembranated models of Chlamydomonas. Rupress Org. 98:97–107. 10.1083/jcb.98.1.97 PMC2112995 6707098

[bib87] Keeling PJ 2019. Combining morphology, behaviour and genomics to understand the evolution and ecology of microbial eukaryotes. Philos Trans R Soc B Biol Sci. 374:1786. 10.1098/rstb.2019.0085 PMC6792444 31587641

[bib88] Keeling PJ , Campo Jd. 2017. Marine protists are not just big bacteria. Curr Biol. 27:R541–9. 28586691 10.1016/j.cub.2017.03.075

[bib89] Kessler DA , Levine H 1993. Pattern formation in *dictyostelium* via the dynamics of cooperative biological entities. Phys Rev E. 48:4801–4. 10.1103/physreve.48.4801 9961163

[bib90] Khona DK , Rao VG, Motiwalla MJ, Varma PCS, Kashyap AR, Das K, Shirolikar SM, Borde L, Dharmadhikari JA, Dharmadhikari AK et al. 2013. Anomalies in the motion dynamics of long-flagella mutants of Chlamydomonas reinhardtii. J Biol Phys. 39:1–14. 23860831 10.1007/s10867-012-9282-8 PMC3532669

[bib91] King JS , Insall RH 2009. Chemotaxis: finding The way forward with dictyostelium. Trends Cell Biol. 19:523–30. 19733079 10.1016/j.tcb.2009.07.004

[bib92] King N , Hittinger CT, Carroll SB 2003. Evolution of key cell signaling and adhesion protein families predates animal origins. Science. 301:361–3. 12869759 10.1126/science.1083853

[bib93] King SJ , Dutcher SK 1997. Phosphoregulation of an inner Dynein arm complex in Chlamydomonas reinhardtii is altered in phototactic mutant strains. J Cell Biol. 136:177–91. 9008712 10.1083/jcb.136.1.177 PMC2132467

[bib94] Kirkegaard JB , Bouillant A, Marron AO, Leptos KC, Goldstein RE 2016. Aerotaxis in the closest relatives of animals. Elife. 5:18109. 10.7554/eLife.18109 PMC5122458 27882869

[bib95] Kirkegaard JB , Goldstein RE 2016. Filter-feeding, near-field flows, and the morphologies of colonial choanoflagellates. Phys Rev E. 94:052401. 27967109 10.1103/PhysRevE.94.052401 PMC6054299

[bib96] Kondo S , Asai R 1995. A reaction–diffusion wave on the skin of the marine angelfish pomacanthus. Nature. 376:765–8. 24547605 10.1038/376765a0

[bib97] Koonin EV 2010. The origin and early evolution of eukaryotes in the light of phylogenomics. Genome Biol. 11:209. 20441612 10.1186/gb-2010-11-5-209 PMC2898073

[bib98] Kuchka MR , Jarvik JW 1987. Short-flagella mutants of chlamydomonas reinhardtii. Genetics. 115:685–91. 17246376 10.1093/genetics/115.4.685 PMC1203101

[bib99] Lane N 2015. The unseen world: reflections On Leeuwenhoek (1677) ‘concerning little animals.’ Phil Trans R Soc B. 370:20140344, 25750239 10.1098/rstb.2014.0344 PMC4360124

[bib100] Larson BT , Garbus J, Pollack JB, Marshall WF 2022. A unicellular walker controlled by a microtubule-based finite-state machine. Curr Biol. 32:3745–3757.e7. 35963241 10.1016/j.cub.2022.07.034 PMC9474717

[bib101] Larson BT , Ruiz-Herrero T, Lee S, Kumar S, Mahadevan L, King N 2020. Biophysical principles of choanoflagellate self-organization. Proc Natl Acad Sci USA. 117:1303–11. 31896587 10.1073/pnas.1909447117 PMC6983409

[bib102] Latty T , Beekman M 2009. Food quality affects search strategy in the acellular slime mould, physarum polycephalum. Behav Ecol. 20:1160–7.

[bib103] Leadbeater BSC 2015. The Choanoflagellates. Cambridge, UK: Cambridge University Press.

[bib104] Lee J , Leonard M, Oliver T, Ishihara A, Jacobson K 1994. Traction forces generated by locomoting keratocytes. J Cell Biol. 127:1957–64. 7806573 10.1083/jcb.127.6.1957 PMC2120302

[bib105] Leeuwenhoek AV . 1677. Observations, communicated to the publisher by Mr. Antony van Leewenhoeck, in a dutch letter of the 9th Octob. 1676. Here english'd: concerning Little animals by him observed in rain-well-sea- and snow water; as also in water wherein pepper had lain infus. Philos Trans R Soc London. 12:821–31.

[bib106] Leeuwenhoek AV . 1700. IV. Part of a letter from Mr Antony Van Leeuwenhoek, concerning the worms in Sheeps livers, gants and animalcula in the excrements of Frogs. Phil Trans R Soc. 22:509–18.

[bib107] Leeuwenhoek AV , Dobell C 1932. Antony van Leeuwenhoek and his “little animals”: being Some account of the father of protozoology and bacteriology and his multifarious discoveries in these disciplines. New York, NY: Brace and Company.

[bib108] Leidy J 1879. Fresh-water rhizopods of North America. Washington, DC: Government Printing Office.

[bib109] Leptos KC , Chioccioli M, Furlan S, Pesci AI, Goldstein RE 2023. Phototaxis of chlamydomonas arises from a tuned adaptive photoresponse shared with multicellular Volvocine green algae. Phys. Rev. E. 107:014404. 36797913 10.1103/PhysRevE.107.014404 PMC7616094

[bib110] Libby E , Ratcliff W, Travisano M, Kerr B 2014. Geometry shapes evolution of early multicellularity. PLoS Comput Biol. 10:e1003803. 25233196 10.1371/journal.pcbi.1003803 PMC4168977

[bib111] Lind PA , Farr AD, Rainey PB 2015. Experimental evolution reveals hidden diversity in evolutionary pathways. Elife. 4:07074. 10.7554/eLife.07074 PMC4395868 25806684

[bib112] Lind PA , Farr AD, Rainey PB 2017. Evolutionary convergence in experimental Pseudomonas populations. ISME J. 11:589. 27911438 10.1038/ismej.2016.157 PMC5322309

[bib113] Lopez-Rios J , Duchesne A, Speziale D, Andrey G, Peterson KA, Germann P, Ünal E, Liu J, Floriot S, Barbey S et al. 2014. Attenuated sensing of SHH by Ptch1 underlies evolution of bovine limbs. Nature. 511:46–51. 24990743 10.1038/nature13289

[bib114] Machemer H 1972. Ciliary activity and the origin of metachrony in Paramecium: effects of increased viscosity. J Exp Biol. 57:57–239. 10.1242/jeb.57.1.239 5075893

[bib115] Machin KE 1963. The control and synchronization of flagellar movement. Proc R Soc London Ser B Biol Sci. 158:88–104.

[bib116] Marbach S , Alim K, Andrew N, Pringle A, Brenner MP 2016. Pruning to increase Taylor dispersion in *physarum polycephalum* networks. Phys Rev Lett. 117:178103. 27824465 10.1103/PhysRevLett.117.178103

[bib117] Marée AF , Hogeweg P 2001. How amoeboids self-organize into a fruiting body: multicellular Coordination in dictyostelium discoideum. Proc Natl Acad Sc. USA. 98:3879–83. 11274408 10.1073/pnas.061535198 PMC31146

[bib118] Marshall WF 2019. Cellular cognition: sequential logic in a giant protist. Curr Biol. 29:R1303–5., 31846675 10.1016/j.cub.2019.10.034 PMC7017933

[bib119] Martin AC , Goldstein B 2014. Apical constriction: themes And variations on a cellular mechanism driving morphogenesis. Development. 141:1987–98. 24803648 10.1242/dev.102228 PMC4011084

[bib120] Matsumoto K , Takagi S, Nakagaki T 2008. Locomotive mechanism of physarum plasmodia based on spatiotemporal analysis of protoplasmic streaming. Biophys J. 94:2492–504. 18065474 10.1529/biophysj.107.113050 PMC2267142

[bib121] Matsumoto K , Ueda T, Kobatake Y 1986. Propagation of phase wave in relation to tactic responses by the plasmodium of *physarum polycephalum*. J Theor Biol. 122:339–45.

[bib122] Matt G , Umen J 2016. Volvox: a simple algal model for embryogenesis, morphogenesis and cellular differentiation. Dev Biol. 419:99–113. 27451296 10.1016/j.ydbio.2016.07.014 PMC5101179

[bib123] McFadden GI 2014. Origin and evolution of plastids and photosynthesis in eukaryotes. Cold Spring Harbor Perspect Biol. 6:a016105. 10.1101/cshperspect.a016105 PMC3970417 24691960

[bib124] Meinhardt H , Gierer A 2000. Pattern formation by local self-activation and lateral inhibition. BioEssays. 22:753–60. 10918306 10.1002/1521-1878(200008)22:8<753::AID-BIES9>3.0.CO;2-Z

[bib125] Mitchell B , Jacobs R, Li J, Chien S, Kintner C 2007. A positive feedback mechanism governs the polarity and motion of motile cilia. Nature. 447:97–101. 17450123 10.1038/nature05771

[bib126] Mogilner A , Manhart A 2016. Agent-based modeling: case Study in cleavage furrow models. MBoC. 27:3379–84. 27811328 10.1091/mbc.E16-01-0013 PMC5221574

[bib127] Mogilner A , Oster G 1996. Cell motility driven by actin polymerization. Biophys J. 71:3030–45. 8968574 10.1016/S0006-3495(96)79496-1 PMC1233792

[bib128] Mogilner A , Oster G 2003. Force generation by Actin polymerization II: the elastic ratchet and tethered Filaments. Biophys J. 84:1591–605. 12609863 10.1016/S0006-3495(03)74969-8 PMC1302730

[bib129] Murray JD , Oster GF 1984. Generation of biological pattern and form. Math Med Biol. 1:51–75. 10.1093/imammb/1.1.51 6600092

[bib130] Murray JDD , Maini PKK, Tranquillo RTT 1988. Mechanochemical models for generating biological pattern and form in development. Phys Rep. 171:59–84.

[bib131] Nagano S 1998. Diffusion-assisted aggregation and synchronization in *dictyostelium discoideum*. Phys Rev Lett. 80:4826–9.

[bib132] Nakagaki T , Yamada H, Ito M 1999. Reaction–Diffusion–Advection model for pattern formation of rhythmic contraction in a giant amoeboid cell of the physarum plasmodium. J Theor Biol. 197:497–506. 10196092 10.1006/jtbi.1998.0890

[bib133] Nakagaki T , Yamada H, Tóth Á 2000. Maze-solving by an amoeboid organism. Nature. 407:470. 11028990 10.1038/35035159

[bib134] Nakagaki T , Yamada H, Tóth Á 2001. Path finding by tube morphogenesis in an amoeboid organism. Biophys Chem. 92:47–52. 11527578 10.1016/s0301-4622(01)00179-x

[bib135] Newman S , Frisch H 1979. Dynamics of skeletal pattern formation in developing chick limb. Science. 205:662–8., 462174 10.1126/science.462174

[bib136] Newman SA , Forgacs G, Muller GB 2006. Before programs: the Physical origination of multicellular forms. Int J Dev Biol. 50:289–99. 16479496 10.1387/ijdb.052049sn

[bib137] Newman SA , Glimm T, Bhat R 2018. The vertebrate limb: an evolving complex of self-organizing systems. Prog Biophys Biophys Chem. 137:12–24. 10.1016/j.pbiomolbio.2018.01.002 29325895

[bib138] Niklas KJ 1988. Biophysical limitations on plant form and evolution. In: Plant Evolutionary Biology. Dordrecht, ND: Chapman and Hall Ltd. p. 185–220.

[bib139] Niklas KJ 1997. Adaptive walks through fitness landscapes for early vascular land plants. Am J Bot. 84:16–25.

[bib140] Niklas KJ 1999. Evolutionary walks through a land plant morphospace. J Exp Bot. 50:39–52.

[bib141] Niklas KJ 2013. Biophysical and size-dependent perspectives on plant evolution. J Exp Bot. 64:4817–27. 23362301 10.1093/jxb/ers379

[bib142] Niklas KJ , Kerchner V 1984. Mechanical and photosynthetic constraints on the evolution of plant shape. Paleobiology. 10:79–101.

[bib143] Nishii I , Ogihara S 1999. Actomyosin contraction of the posterior hemisphere is required for inversion of the Volvox embryo. Development. 126:2117–27. 10207137 10.1242/dev.126.10.2117

[bib144] Nishii I , Ogihara S, Kirk DL 2003. A Kinesin, InvA, plays an essential role in volvox morphogenesis. Cell. 113:743–53. 12809605 10.1016/s0092-8674(03)00431-8

[bib145] Odell GM , Oster G, Alberch P, Burnside B 1981. The mechanical basis of morphogenesis. I. Epithelial folding and invagination. Dev Biol. 85:446–62. 7196351 10.1016/0012-1606(81)90276-1

[bib146] Oettmeier C , Brix K, Döbereiner H-G 2017. *Physarum polycephalum*—A New take on a classic model system. J Phys D Appl Phys. 50:413001.

[bib147] Okajima A , Kinosita H 1966. Ciliary activity and coordination in Euplotes eurystomus-I. Effect of microdissection of neuromotor fibres. Comp Biochem Physiol. 19:115–31.

[bib148] Oster GF , Murray JD, Harris AK 1983. Mechanical aspects of mesenchymal morphogenesis. J Embryol Exp Morphol. 78:83–125. 6663234

[bib149] Oster GF , Shubin N, Murray JD, Alberch P 1988. Evolution and morphogenetic rules: the shape of the vertebrate limb in ontogeny and phylogeny. Evolution (N Y). 42:862–84. 10.1111/j.1558-5646.1988.tb02508.x 28581162

[bib150] Párducz B 1967. Ciliary Movement and Coordination in Ciliates. Int Rev Cytol. 21:91–128. 4961084 10.1016/s0074-7696(08)60812-8

[bib151] Petrov V , Gáspár V, Masere J, Showalter K 1993. Controlling chaos in the Belousov—Zhabotinsky reaction. Nature. 361:240–3.

[bib152] Polin M , Tuval I, Drescher K, Gollub JP, Goldstein RE 2009. Chlamydomonas swims with two “gears” in a eukaryotic version of run-and-tumble locomotion. Science. 325:487–90. 19628868 10.1126/science.1172667

[bib153] Pollard TD 2010. Mechanics of cytokinesis in eukaryotes. Curr Opin Cell Biol. 22:50–6. 20031383 10.1016/j.ceb.2009.11.010 PMC2871152

[bib154] Pollard TD , Cooper JA 2009. Actin, a central player in cell shape and movement. Science. 326:1208–12. 19965462 10.1126/science.1175862 PMC3677050

[bib155] Purcell EM 1977. Life at low Reynolds number. Am J Phys. 45:3–11.

[bib156] Quaranta G , Aubin-Tam ME, Tam D 2015. Hydrodynamics versus intracellular coupling in the synchronization of eukaryotic flagella. Phys Rev Lett. 115:238101. 26684142 10.1103/PhysRevLett.115.238101

[bib157] Rajan D , Makushok T, Kalish A, Acuna L, Bonville A, Almanza KC, Garibay B, Tang E, Voss M, Lin A et al. 2023. Single-cell analysis of habituation in Stentor coeruleus. Curr Biol. 33:241–251.e4. 36435177 10.1016/j.cub.2022.11.010 PMC9877177

[bib158] Ramirez-San Juan GR , Mathijssen AJTM, He M, Jan L, Marshall W, Prakash M. 2020. Multi-scale spatial heterogeneity enhances particle clearance in airway ciliary arrays. Nat Phys. 16:958–64. 35937969 10.1038/s41567-020-0923-8 PMC9355487

[bib159] Raup DM 1967. Geometric analysis of shell coiling: coiling in ammonoids. JPaleo. 41:43–65.

[bib160] Rauzi M , Krzic U, Saunders TE, Krajnc M, Ziherl P, Hufnagel L, Leptin M 2015. Embryo-scale tissue mechanics during Drosophila gastrulation movements. Nat Commun. 6:8677. 26497898 10.1038/ncomms9677 PMC4846315

[bib161] Reid CR , Beekman M, Latty T, Dussutour A 2013. Amoeboid organism uses extracellular secretions to make smart foraging decisions. Behav Ecol. 24:812–8.

[bib162] Reid CR , Latty T, Dussutour A, Beekman M 2012. Slime mold uses an externalized spatial “memory” to navigate in complex environments. Proc Natl Acad Sci USA. 109:17490–4. 23045640 10.1073/pnas.1215037109 PMC3491460

[bib163] Reid CR , MacDonald H, Mann RP, Marshall JAR, Latty T, Garnier S 2016. Decision-making without a brain: how An amoeboid organism solves the two-armed bandit. J R Soc Interface. 13:20160030. 27278359 10.1098/rsif.2016.0030 PMC4938078

[bib164] Richardson E , Zerr K, Tsaousis A, Dorrell RG, Dacks JB 2015. Evolutionary cell biology: functional Insight from “endless forms most beautiful”. MBoC. 26:4532–8. 26668171 10.1091/mbc.E14-10-1433 PMC4678011

[bib165] Richter DJ , Fozouni P, Eisen MB, King N 2018. Gene family innovation, conservation and loss on the animal stem lineage. Elife. 7:34226. 10.7554/eLife.34226 PMC6040629 29848444

[bib166] Riedel IH , Kruse K, Howard J 2005. A self-organized vortex array of hydrodynamically entrained sperm cells. Science. 309:300–3. 16002619 10.1126/science.1110329

[bib167] Roper M , Dayel MJ, Pepper RE, Koehl MAR. 2013. Cooperatively generated stresslet flows supply fresh fluid to multicellular choanoflagellate colonies. Phys Rev Lett. 110:228104. 23767751 10.1103/PhysRevLett.110.228104

[bib168] Ruiz-Trillo I , Roger AJ, Burger G, Gray MW, Lang BF 2008. A phylogenomic investigation into the origin of metazoa. Mol Biol Evol. 25:664–72. 18184723 10.1093/molbev/msn006

[bib169] Salazar-Ciudad I 2012. Tooth patterning and evolution. Curr Opin Genet Dev. 22:585–92. 23266218 10.1016/j.gde.2012.10.006

[bib170] Salazar-Ciudad I , Jernvall J 2002. A gene network model accounting for development and evolution of mammalian teeth. Proc Natl Acad Sci USA. 99:8116–20. 12048258 10.1073/pnas.132069499 PMC123030

[bib171] Salazar-Ciudad I , Jernvall J 2010. A computational model of teeth and the developmental origins of morphological variation. Nature. 464:583–6. 20220757 10.1038/nature08838

[bib172] Sebe-Pedros A , de Mendoza A, Lang BF, Degnan BM, Ruiz-Trillo I 2011. Unexpected repertoire of metazoan transcription factors in the unicellular holozoan capsaspora owczarzaki. Mol Biol Evol. 28:1241–54. 21087945 10.1093/molbev/msq309 PMC4342549

[bib173] Shaffer BM 1975. Secretion of cyclic AMP induced by cyclic AMP in the cellular slime mould dictyostelium discoideum. Nature. 255:549–52. 167286 10.1038/255549a0

[bib174] Sheth R , Marcon L, Bastida MF, Junco M, Quintana L, Dahn R, Kmita M, Sharpe J, Ros MA 2012. Hox genes regulate digit patterning by controlling the wavelength of a Turing-type mechanism. Science. 338:1476–80. 23239739 10.1126/science.1226804 PMC4486416

[bib175] Short MB , Solari CA, Ganguly S, Powers TR, Kessler JO, Goldstein RE 2006. Flows driven by flagella of multicellular organisms enhance long-range molecular transport. Proc Natl Acad Sci USA. 103:8315–9. 16707579 10.1073/pnas.0600566103 PMC1482491

[bib176] Shyer AE , Rodrigues AR, Schroeder GG, Kassianidou E, Kumar S, Harland RM 2017. Emergent cellular self-organization and mechanosensation initiate follicle pattern in the avian skin. Science. 357:811–5. 28705989 10.1126/science.aai7868 PMC5605277

[bib177] Sick S , Reinker S, Timmer J, Schlake T 2006. WNT and DKK determine hair follicle spacing through a reaction-diffusion mechanism. Science. 314:1447–50. 17082421 10.1126/science.1130088

[bib178] Sineshchekov OA , Govorunova EG 1999. Rhodopsin-mediated photosensing in green flagellated algae. Trends Plant Sci. 4:58–63. 10234274 10.1016/s1360-1385(98)01370-3

[bib179] Smyth RD , Berg HC 1982. Change in flagellar beat frequency of Chlamydomonas in response to light. Cell Motility. 2:211–5. 10.1002/cm.970020740 7100179

[bib180] Soh AWJ , Van Dam TJP, Stemm-Wolf AJ, Pham AT, Morgan GP, O'Toole ET, Pearson CG. 2020. Ciliary force-responsive striated fibers promote basal body connections and cortical interactions. J Cell Biol. 219:e201904091. 31740506 10.1083/jcb.201904091 PMC7039215

[bib181] Soh AWJJ , Woodhams LG, Junker AD, Enloe CM, Noren BE, Harned A, Westlake CJ, Narayan K, Oakey JS, Bayly PV et al. 2022. Intracellular connections between basal bodies promote the coordinated behavior of motile cilia. MBoC. 33:1–14. 10.1091/mbc.E22-05-0150 PMC9582806 35767367

[bib182] Solari CA , Drescher K, Ganguly S, Kessler JO, Michod RE, Goldstein RE 2011. Flagellar phenotypic plasticity in volvocalean algae correlates with Peclet number. J R Soc Interface. 8:1409–17. 21367778 10.1098/rsif.2011.0023 PMC3163421

[bib183] Solari CA , Ganguly S, Kessler JO, Michod RE, Goldstein RE 2006. Multicellularity and the functional interdependence of motility and molecular transport. Proc Natl Acad Sci USA. 103:1353–8. 16421211 10.1073/pnas.0503810103 PMC1360517

[bib184] Spiers AJ 2014. A mechanistic explanation linking adaptive mutation, niche change, and fitness advantage for the wrinkly spreader. Int J Evol Biol. 2014:1. 10.1155/2014/675432 PMC3914426 24551477

[bib185] Spiers AJ , Kahn SG, Bohannon J, Travisano M, Rainey PB 2002. Adaptive divergence in experimental populations of Pseudomonas fluorescens. I. Genetic and phenotypic bases of wrinkly spreader fitness. Genetics. 161:33–46. 12019221 10.1093/genetics/161.1.33 PMC1462107

[bib186] Sriskanthadevan S , Zhu Y, Manoharan K, Yang C, Siu C-H, Siu C-H 2011. The cell adhesion molecule DdCAD-1 regulates morphogenesis through differential spatiotemporal expression in dictyostelium discoideum. Development. 138:2487–97. 21561987 10.1242/dev.060129

[bib187] Tam D , Hosoi AE 2011. Optimal feeding and swimming gaits of biflagellated organisms. Proc Natl Acad Sci USA. 108:1001–6. 21199951 10.1073/pnas.1011185108 PMC3024673

[bib188] Taylor CV 1921. Demonstration of the function of the neuromotor apparatus in euplotes by the method of microdissection. Naturwissenschaften. 9:910.

[bib189] Tero A , Takagi S, Saigusa T, Ito K, Bebber DP, Fricker MD, Yumiki K, Kobayashi R, Nakagaki T 2010. Rules for biologically inspired adaptive network design. Science. 327:439–42. 20093467 10.1126/science.1177894

[bib190] Thompson DW 1917. On Growth and Form. Cambridge, UK: Cambridge University Press.

[bib191] Trinh MK , Wayland MT, Prabakaran S 2019. Behavioural analysis of single-cell aneural ciliate, Stentor roeseli, using machine learning approaches. J R Soc Interface. 16:20190410, 31795860 10.1098/rsif.2019.0410 PMC6936043

[bib192] Tsuda S , Aono M, Gunji Y-P 2004. Robust and emergent Physarum logical-computing. Biosystems. 73:45–55. 14729281 10.1016/j.biosystems.2003.08.001

[bib193] Turing AM 1952. The chemical basis of morphogenesis. Philos Trans R Soc Lond B Biol Sci. 237:37–72. 10.1098/rstb.2014.0218 PMC4360114 25750229

[bib194] Tweedy L , Insall RH 2020. Self-generated gradients yield exceptionally robust steering cues. Front Cell Dev Biol. 8:133. 32195256 10.3389/fcell.2020.00133 PMC7066204

[bib195] Tweedy L , Knecht DA, Mackay GM, Insall RH 2016. Self-generated chemoattractant gradients: attractant depletion extends the range and robustness of chemotaxis. PLOS Biol. 14:e1002404. 26981861 10.1371/journal.pbio.1002404 PMC4794234

[bib196] Tweedy L , Thomason PA, Paschke PI, Martin K, Machesky LM, Zagnoni M, Insall RH 2020. Seeing around corners: cells solve mazes and respond at a distance using attractant breakdown. Science. 369:eaay9792. 32855311 10.1126/science.aay9792

[bib197] Tyson JJ 1979. Oscillations, bistability, and echo waves in models of the Belousov-Zhabotinskii reaction. Annals of the New York Academy of Sciences. 316:279–95.

[bib198] Ueki N , Matsunaga S, Inouye I, Hallmann A 2010. How 5000 independent rowers coordinate their strokes in order to row into the sunlight: phototaxis in the multicellular green alga Volvox. BMC Biol. 8:103. 20663212 10.1186/1741-7007-8-103 PMC2920248

[bib199] Ueki N , Nishii I 2009. Controlled enlargement of the glycoprotein vesicle surrounding a volvox embryo requires the InvB nucleotide-sugar transporter and is required for normal morphogenesis. Plant Cell. 21:1166–81. 19346464 10.1105/tpc.109.066159 PMC2685634

[bib200] Ueki N , Wakabayashi Ki. 2018. Detergent-extracted Volvox model exhibits an anterior–posterior gradient in flagellar Ca2+ sensitivity. Proc Natl Acad Sci USA. 115:E1061–68. 29311312 10.1073/pnas.1715489115 PMC5798354

[bib201] Verasztó C , Ueda N, Bezares-Calderón LA, Panzera A, Williams EA, Shahidi R, Jékely G 2017. Ciliomotor circuitry underlying whole-body coordination of ciliary activity in the *Platynereis larva*. Elife. 6:26000. 10.7554/eLife.26000 PMC5531833 28508746

[bib202] Viamontes GI , Kirk DL 1977. Cell shape changes and the mechanism of inversion in Volvox. J Cell Biol. 75:719–30. 925078 10.1083/jcb.75.3.719 PMC2111588

[bib203] Vijayraghavan DS , Davidson LA 2017. Mechanics of neurulation: from classical to current perspectives on the physical mechanics that shape, fold, and form the neural tube. Birth Defects Research. 109:153–68. 27620928 10.1002/bdra.23557 PMC9972508

[bib204] Vogel D , Dussutour A 2016. Direct transfer of learned behaviour via cell fusion in non-neural organisms. Proc R Soc B Biol Sci. 283:1845. 10.1098/rspb.2016.2382 PMC5204175 28003457

[bib205] von Dassow M , Davidson LA 2007. Variation and robustness of the mechanics of gastrulation: the role of tissue mechanical properties during morphogenesis. Birth Defect Res C. 81:253–69. 10.1002/bdrc.20108 18228257

[bib206] Wan KY 2018. Coordination of eukaryotic cilia and flagella. Essays Biochem. 62:829–38. 30464007 10.1042/EBC20180029 PMC6281475

[bib207] Wan KY , Goldstein RE 2016. Coordinated beating of algal flagella is mediated by basal coupling. Proc Natl Acad Sci USA. 113:E2784–93. 27140605 10.1073/pnas.1518527113 PMC4878519

[bib208] Wan KY , Jékely G 2021. Origins of eukaryotic excitability. Phil Trans R Soc B. 376:20190758. 33487111 10.1098/rstb.2019.0758 PMC7935092

[bib209] Weijer CJ 2004. Dictyostelium morphogenesis. Curr Opin Genet Dev. 14:392–8. 15261655 10.1016/j.gde.2004.06.006

[bib210] Williams TA 2014. Evolution: rooting the eukaryotic tree of life. Curr Biol. 24:R151–2. 24556435 10.1016/j.cub.2014.01.026

[bib211] Woolley DM , Crockett RF, Groom WDI, Revell SG 2009. A study of synchronisation between the flagella of bull spermatozoa, with related observations. J Exp Biol. 212:2215–23. 19561211 10.1242/jeb.028266

[bib212] Yamada H , Nakagaki T, Ito M 1999. Pattern formation of a reaction-diffusion system with self-consistent flow in the amoeboid organism *physarum plasmodium*. Phys Rev E. 59:1009–14.

[bib213] Yamaguchi M , Yoshimoto E, Kondo S 2007. Pattern regulation in the stripe of zebrafish suggests an underlying dynamic and autonomous mechanism. Proc Natl Acad Sci USA. 104:4790–3. 17360399 10.1073/pnas.0607790104 PMC1820822

[bib214] Zhabotinsky AM 1964. Periodical process of oxidation of malonic acid solution. Biofisika. 9:306–11. 14206238

